# Short-chain fatty acids of various lengths differentially inhibit *Klebsiella pneumoniae* and *Enterobacteriaceae* species

**DOI:** 10.1128/msphere.00781-23

**Published:** 2024-02-02

**Authors:** Kai Chirng Chang, Niranjan Nagarajan, Yunn-Hwen Gan

**Affiliations:** 1 Yong Loo Lin School of Medicine, National University of Singapore, Singapore, Singapore; 2 Genome Institute of Singapore (GIS), Agency for Science, Technology and Research (A*STAR), Singapore, Singapore; 3 Department of Biochemistry, Infectious Diseases Translational Research Programme, Yong Loo Lin School of Medicine, National University of Singapore, Singapore, Singapore; University of Michigan-Ann Arbor, Ann Arbor, Michigan, USA

**Keywords:** *Klebsiella pneumoniae*, colonization, short-chain fatty acids, *Enterobacteriaceae*, intestine, bacteriostatic

## Abstract

The gut microbiota is inextricably linked to human health and disease. It can confer colonization resistance against invading pathogens either through niche occupation and nutrient competition or via its secreted metabolites. Short-chain fatty acids (SCFA) are the primary metabolites in the gut as a result of dietary fiber fermentation by the gut microbiota. In this work, we demonstrate that the interaction of single-species gut commensals on solid media is insufficient for pathogen inhibition, but supernatants from monocultures of these commensal bacteria enriched in acetate confer inhibition against anaerobic growth of the enteric pathogen *Klebsiella pneumoniae*. The three primary SCFAs (acetate, propionate, and butyrate) strongly inhibit the intestinal commensal *Escherichia coli* Nissle as well as a panel of enteric pathogens besides *K. pneumoniae* at physiological pH of the cecum and ascending colon. This inhibition was significantly milder on anaerobic gut commensals *Bacteroides thetaiotaomicron* and *Bifidobacterium adolescentis* previously demonstrated to be associated with microbiota recovery after antibiotic-induced dysbiosis. We describe a general suppression of bacterial membrane potential by these SCFAs at physiological cecum and ascending colonic pH. Furthermore, the strength of bacterial inhibition increases with increasing alkyl chain length. Overall, the insights gained in this study shed light on the potential therapeutic use of SCFAs for conferring colonization resistance against invading pathogens in a dysbiotic gut.

**IMPORTANCE:**

Rising antimicrobial resistance has made treatment of bacterial infections increasingly difficult. According to the World Health Organization, it has become a burgeoning threat to hospital and public health systems worldwide. This threat is largely attributed to the global rise of carbapenem-resistant *Enterobacteriaceae* in recent years, with common hospital-acquired pathogens growing increasingly resistant to last-line antibiotics. Antibiotics disrupt the homeostatic balance of the gut microbiota, resulting in the loss of colonization resistance against enteric pathogens. This work describes the ability of short-chain fatty acids (SCFAs) produced by gut microbiota to be effective against a wide panel of enteric pathogens without major impact on common gut commensal species. We also demonstrate a previously undescribed link between alkyl chain length and antibacterial effects of SCFAs. SCFAs, thus, hold promise as an alternative therapeutic option leveraging on the antimicrobial activity of these endogenously produced gut metabolites without disrupting gut microbiota homeostasis.

## INTRODUCTION

Colonization resistance is the phenomenon of preventing pathogen or pathobiont invasion within established microbial communities and niches (1). It is best documented in the context of the gut where the colon contains 10^13^–10^14^ microbial cells intricately associated with host health and function via interactions with host epithelia and within microbial communities (2). A healthy gut microbiome provides protection against the colonization of pathogenic enteric species known to harbor multiple drug resistances (3). It can mediate colonization resistance via nutrient competition and production of bacteriocins, toxins, or inhibitory metabolites such as bile acids or short-chain fatty acids (SCFAs). Colonization resistance can also be exerted via indirect mechanisms such as the mucus barrier, which limits pathogen interaction with underlying epithelial cells, and the maintenance of intestinal hypoxia to limit oxygen and prevent expansion of facultative anaerobe pathogens (4). It has been established that antibiotic usage can induce dysbiosis and disrupt colonization resistance to opportunistic pathogens. Antibiotic treatment, particularly clindamycin, has long been associated with the greatest risk of developing *Clostridium difficile* infections (5–7). Patients who underwent antibiotic treatment also developed systemic *Salmonella enterica* Typhimurium infections shortly after (8, 9). Streptomycin-induced disturbance to the gut flora also increased *S. enterica* serovar Typhimurium murine gut colonization by 100,000-fold (10). Similarly, metronidazole administration to mice has been reported to facilitate *Citrobacter rodentium* adhesion to the colonic mucus layer and exacerbates severity of colitis development (11).

In a metagenome-wide association study, Chng et al.assembled data based on gut microbiome profiling of individuals from four different countries in three continents from a range of age groups who had undergone treatment with different classes of antibiotics (12). Based on the post-antibiotic gut microbiome diversity as their selected metric for recovery, individuals were stratified into “recoverers” or “non-recoverers.” From the recoverers, they identified 21 recovery-associated bacteria, henceforth termed RABs, where six of the strains were enriched in three out of the four total cohorts. In a proof of concept experiment, two of the RABs *Bacteroides thetaiotaomicron* and *Bifidobacterium adolescentis* were administered via oral gavage to mice, which underwent 5 days of antibiotic-mediated microbiome disruption. It was found that combinatorial administration of both these species increased bacterial biomass and diversity to a greater degree than mice, which recovered naturally without probiotic intervention.

*B. thetaiotaomicron* is proposed to be a keystone species for microbiome recovery (12) and is an important human symbiont (13). It belongs to the *Bacteroides* genus, which makes up approximately 30% of the human gut and contains 88 polysaccharide utilizing locus (PULs) encoding specialized starch utilization (sus) genes encompassing 18% of its entire genome (13). Its metabolic diversity confers the ability unique to only a limited subset of anaerobes in the gut microbiota to redirect metabolism from diet-derived complex carbohydrates to cleavage of host mucin O-glycans (14). *B. adolescentis* belongs to the Actinobacteria phyla and is the first to colonize the gut of breast-fed infants due to its specialized metabolism of human milk oligosaccharides (HMOs), before it stabilizes to 3%–6% in the adult gut where a reduction in Bifidobacterium levels has been associated with several disease states (15). Glycoside hydrolases are the main enzymes prevalent in Bifidobacterium, which hydrolyzes glycosidic bonds from an extensive range of complex sugars and was described to be a tertiary colonizer due to its heavy reliance on other primary species or diet-derived carbon sources (12). *B. thetaiotaomicron* is a primary keystone species exhibiting direct colonization resistance by nutrient competition and niche occupation, as well as indirectly via the production of antimicrobial peptides such as the C type lectin RegIIIγ (16). Its ability to break down dietary fiber can help trigger a downstream chain of cross-feeding interactions to facilitate the expansion of dietary carbohydrate-degrading gut anaerobes, which do not possess mucin degradative properties such as *B. adolescentis*.

In a longitudinal study on the microbiome dynamics of patients colonized with carbapenemase-producing Enterobacteriaceae (CPE), spontaneous decolonization of CPE occurred over a year without intervention (17). The microbiota of patients who underwent decolonization exhibited distinct ecological shifts with restoration of microbial diversity and seven key commensals including *Bacteroides* and *Bifidobacterium* species. These CPE were mainly *Escherichia coli* and *Klebsiella pneumoniae* with similar antibiotic-resistant profiles and worryingly, shared a pKPC2 plasmid harboring the *bla*_KPC-2_ carbapenemase gene between them. Among the CPE, surveillance studies find *K. pneumoniae*, *Enterobacter cloacae*, and *E. coli* to be the predominant species carrying the most number of carbapenem-resistant *Enterobacteriaceae* plasmids (18, 19). In recent years, there has been a convergence of both carbapenem-resistant and hypervirulent *K. pneumoniae* (hvKP) in Singapore (20), China, Brazil, and the United Kingdom (21, 22), which is a great cause of concern. As hvKP can cause disease in healthy individuals, strategies to prevent transmission of these hypervirulent and carbapenem-resistant bacteria are critical.

The route of transmission and pathogenesis of *K. pneumoniae* is via the fecal-oral route. Upon ingestion, it passes through the gastrointestinal (GI) tract to first establish colonization in the gut (23). Therefore, establishing colonization resistance against *K. pneumoniae* is important for preventing the formation of a reservoir of antibiotic resistance genes (ARG) in the gut that can give rise to transmission. Based on previous work showing that colonization of *B. thetaiotaomicron* and *B. adolescentis* could enhance microbiome recovery (12), we first determined whether these commensals have direct inhibitory effects on hvKP that could contribute to colonization resistance. We found that the inhibitory effects on hvKP are primarily mediated by SCFAs under physiological pH on various *Enterobacteriaceae* species, without major impact on key gut commensals.

## MATERIALS AND METHODS

### Bacterial strains and growth conditions

*K. pneumoniae*, *E. cloacae*, *S. enterica* serovar Typhimurium, and *E. coli* strains were grown in Lysogeny Broth (LB, Invitrogen) and enumerated for CFU counts on LB agar (LBA) plates. *B. thetaiotaomicron*, *B. adolescentis*, and *B. uniformis* were grown and maintained as liquid cultures in reinforced clostridial medium (RCM) and enumerated for CFU on reinforced clostridial agar (RCA) plates. Strain details are in Table 1.

**TABLE 1:** Bacterial strains used in this study

Bacterial strain	Description	Reference
*Klebsiella pneumoniae* SGH10	Hypervirulent clinical isolate, hypermucoviscous, K1 serotype	(24)
*Escherichia coli* Nissle 1917	K-5 intestinal probiotic used in the commercial product Mutaflor for treatment of inflammatory bowel diseases	(25)
*Escherichia coli* MG1655	K-12 wild-type laboratory strain and known intestinal commensal	(26)
*Enterobacter cloacae* ATCC13047	Opportunistic nosocomial pathogen, American Type Culture Collection (ATCC) strain 13047	(27)
*Salmonella enterica* serovar Typhimurium LT2	Invasive pathogen of the gastrointestinal tract, ATCC strain LT2	(28)
*Bacteroides thetaiotaomicron* ATCC29148	Anaerobic gut commensal isolated from human feces, ATCC type strain 29148	(12)
*Bifidobacterium adolescentis* DSM20083	Anaerobic gut commensal isolated from adult human intestinal tract, Deutsche Sammlung von Mikroorganismen und Zelkulturen GmbH (DSM) type strain 20083	(12)

### Competition assays between anaerobes and *K. pneumoniae*

Overnight *K. pneumoniae* SGH10 (hereafter named SGH10) cultures grown in LB and stationary phase *B. thetaiotaomicron*, *B. adolescentis*, and *B. uniformis* cultures maintained in liquid RCM were measured for OD_600_ and mixed together at a ratio of 100:1 anaerobe to SGH10. Then, 25 µL of the competition mix as well as the single bacterial controls of SGH10 and the respective anaerobes was spotted onto nitrocellulose membranes in triplicates on four sets of RCA plates—each corresponding to the duration of incubation of 24, 48, 72, or 96 hours. Single bacterial controls were included to account for growth rate differences between SGH10 and the anaerobes. The plates were incubated at 37°C in a GasPak EZ container (BD) containing GasPak EZ Anaerobe Container System Indicator Sachets to establish an anaerobic environment throughout the course of the experiment. Every 24 hours, the membranes were washed in 3 mL of 1× PBS and serially diluted before relevant dilutions were plated out on 100-µg/mL carbenicillin LBA plates and incubated aerobically to obtain SGH10 counts; and 10-µg/mL gentamicin plates were incubated anaerobically to obtain anaerobe counts. This procedure was repeated until the 96th hour.

### *K. pneumoniae* anaerobic growth in spent supernatants of *B. thetaiotaomicron* and *B. adolescentis*

RCM growth media for anaerobe cultivation were equilibrated overnight in a Don Whitley anaerobic chamber prior to experimental use. *B. thetaiotaomicron* and *B. adolescentis* were sub-cultured in RCM for growth until stationary phase for 3 days. The bacterial supernatants were collected, filtered through a 0.22-µm filter, and pH-adjusted to pH 7.0 and pH 5.8. The supernatants (50% [vol/vol]) were prepared by adding an equal volume of RCM adjusted to pH 7.0 or pH 5.8 into spent supernatants at pH 7.0 or 5.8. Overnight culture of SGH10 was diluted to a final OD_600_ of 0.01 (1.0 × 10^7^ CFU/mL) in RCM media pH-adjusted controls and 50% and 100% spent supernatants of *B. thetaiotaomicron* or *B. adolescentis* at pH 7.0 and 5.8, respectively, and was incubated under anaerobic conditions. An OD_600_ of SGH10 was taken 48 hours later using a microplate reader.

### Gas chromatography-mass spectrometry quantification of SCFAs in spent supernatants of *B. thetaiotaomicron* and *B. adolescentis*

Supernatant samples were prepared in an anaerobic chamber, filter-sterilized, and frozen at −80°C until gas chromatography-mass spectrometry (GC-MS) analysis by PANOMIX Singapore. The analysis was performed on trace 1600 gas chromatograph (Thermo Fisher Scientific, USA). The GC was fitted with a capillary column Agilent HP-INNOWAX (30 m × 0.25 mm ID × 0.25 µm), and helium was used as the carrier gas at 1 mL/min. Mass spectrometric detection of metabolites was performed on ISQ 7610 (Thermo Fisher Scientific, USA) with electron impact ionization mode.

### *In vitro* SCFA inhibition

Short-chain fatty acids sodium acetate (10, 50, and 100 mM; Merck), sodium butyrate (Sigma Aldrich), and sodium propionate (Sigma Aldrich) were prepared in LB, and the various SCFA-supplemented media were titrated to pH 5.75 and pH 7.00 with 1 M hydrochloric acid (HCl) or sodium hydroxide (NaOH) and filtered through a 0.22-µm filter (Millipore). Overnight bacterial cultures were diluted to a final OD_600_ of 0.01 (1.0 × 10^7^ CFU/mL), where OD_600_ of 1 = 1.0 × 10^9^ CFU/mL, into LB alone and LB + SCFA at the various pH and SCFA concentrations. Cultures were grown at 37°C with shaking at 150 rpm before overnight OD_600_ was measured with a spectrophotometer.

### Membrane potential DiOC_2_(3) assay

Overnight bacterial cultures were diluted to OD_600_ of 0.2, pelleted, and resuspended in 30 µM of diethyloxacarbocyanine [DiOC_2_(3)] dye, and incubated at 37°C for 30 minutes. Samples were pelleted, washed twice, and resuspended in 1× PBS containing 0.4% glucose pH-balanced to pH 5.75 or pH 7.0. Bacterial samples (100 µL) were added to 96-well black bottom plates (Corning) and 100 µL of 1× PBS 0.4% glucose controls at pH 5.75 or 7.0, or 50 and 100 mM of sodium acetate, sodium propionate, and sodium butyrate at pH 5.75 or 7.0 were added to wells containing the respective pH-controlled bacterial samples. Red (λ_EX_ 488 nm/λ_EM_ 630 nm) and green (λ_EX_ 488 nm/λ_EM_ 530 nm) fluorescence was read for 2 hours at 15-minute intervals using a microplate reader (Tecan). Membrane potential was calculated as red/green fluorescence emission ratio (λ_EM_ 630 nm/λ_EM_ 530 nm).

### Growth curves

*K. pneumoniae* SGH10 was grown in LB with 50 and 100 mM of sodium acetate, sodium propionate, sodium butyrate, crotonic acid (Sigma), or sodium hexanoate (Sigma), and in LB alone in a 96-well clear bottom plate (Corning). OD_600_ was read every 2 hours with a Tecan Spark 10M plate-reader (Tecan) for a duration of at least 96 hours. 2-(N-morpholino)ethanesulfonic acid (MES) buffer (100 mM; Sigma) was added to all conditions for maintenance of constant pH throughout the growth duration.

### Statistical analysis

All data are presented as mean ± SD. Comparisons between groups were performed using unpaired Student’s *t*-test with Welch’s correction in the GraphPad Prism version 8.0 software. A *P* value of <0.05 was considered statistically significant.

## RESULTS

### *Bacteroides* and *Bifidobacterium* do not directly inhibit *K. pneumoniae* growth

To determine whether gut commensals directly inhibit *K. pneumoniae*, pairwise competition assays were carried out between a hypervirulent *K. pneumoniae* strain SGH10 and anaerobes *B. thetaiotaomicron*, *B. adolescentis*, and *Bacteroides uniformis*, which were species found in multiple patient cohorts to be associated with post-antibiotic recovery (12).

**Fig 1:**
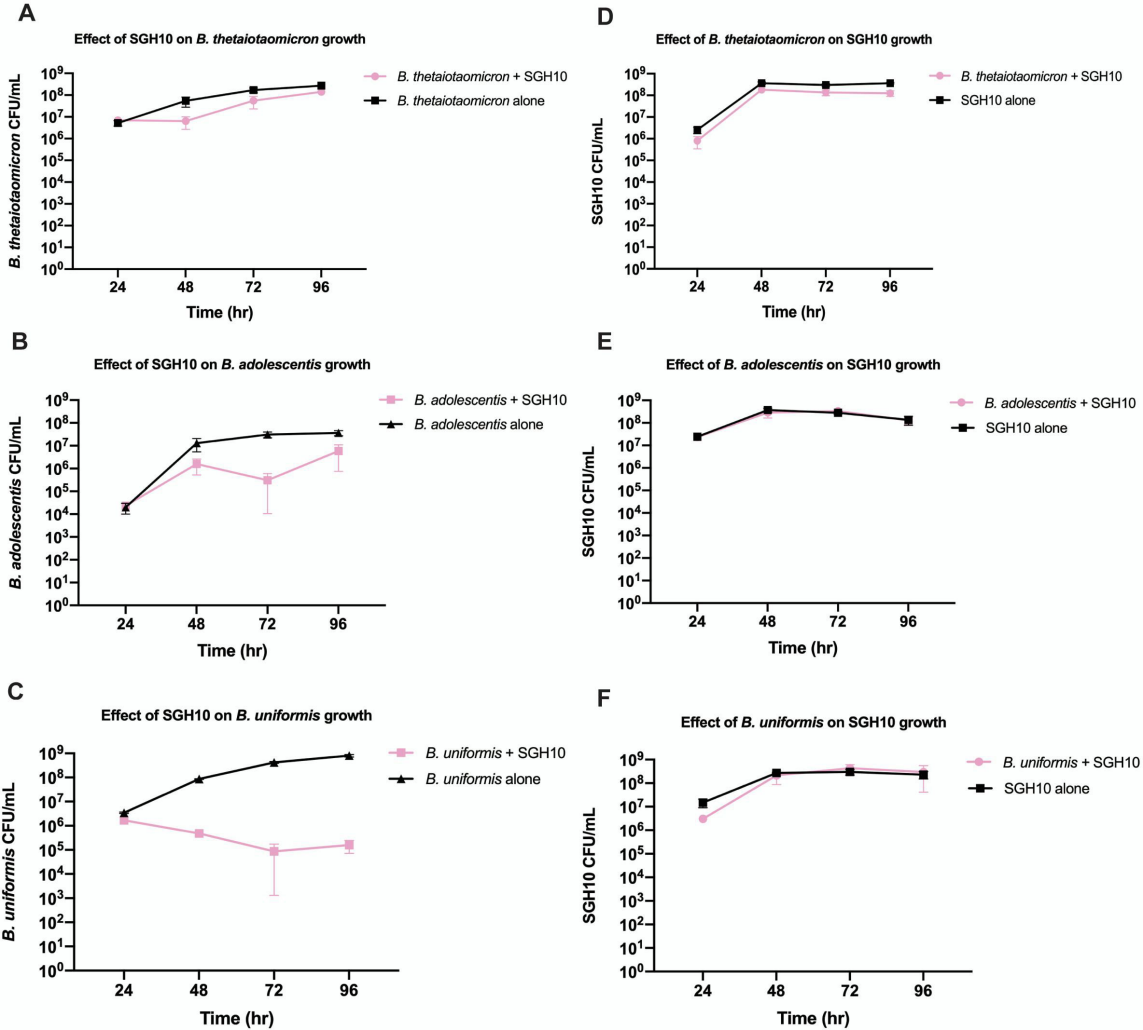
Enumerated CFU counts of SGH10 and gut commensals grown with or without the presence of each other over 96 hours of anaerobic incubation at 37°C on solid RCA. (**A**) *B. thetaiotaomicron*, (**B**) *B. adolescentis*, and (**C**) *B. uniformis* grown with or without SGH10, and corresponding CFUs of SGH10 grown with or without (**D**) *B. thetaiotaomicron*, (**E**) *B. adolescentis*, and (**F**) *B. uniformis*. Independent experiments have been repeated with similar observations.

To account for growth rate disparities, anaerobes were added at 100 times higher than *K. pneumoniae* SGH10. However, instead of the gut commensals inhibiting *K. pneumoniae* SGH10, SGH10 was observed to inhibit the growth of these gut commensals by various degrees. Growth of *B. thetaiotaomicron* in the presence of SGH10 was relatively unperturbed as compared to its growth as a monoculture (Fig. 1A). *B. adolescentis* was inhibited by SGH10 at 48 and 72 hours, but this inhibition was reversed by the end of the experiment as seen by the decrease in the gap between *B. adolescentis* as a monoculture and in the competition mix from 72 to 96 hours (Fig. 1B). *B. uniformis* experienced very persistent growth inhibition by SGH10 across all timepoints throughout the course of the experiment (Fig. 1C). On the other hand, growth of SGH10 was unaffected by the presence of all three gut anaerobes (Fig. 1D through F).

### Supernatants of *B. thetaiotaomicron* and *B. adolescentis* inhibit *K. pneumoniae*

**Fig 2:**
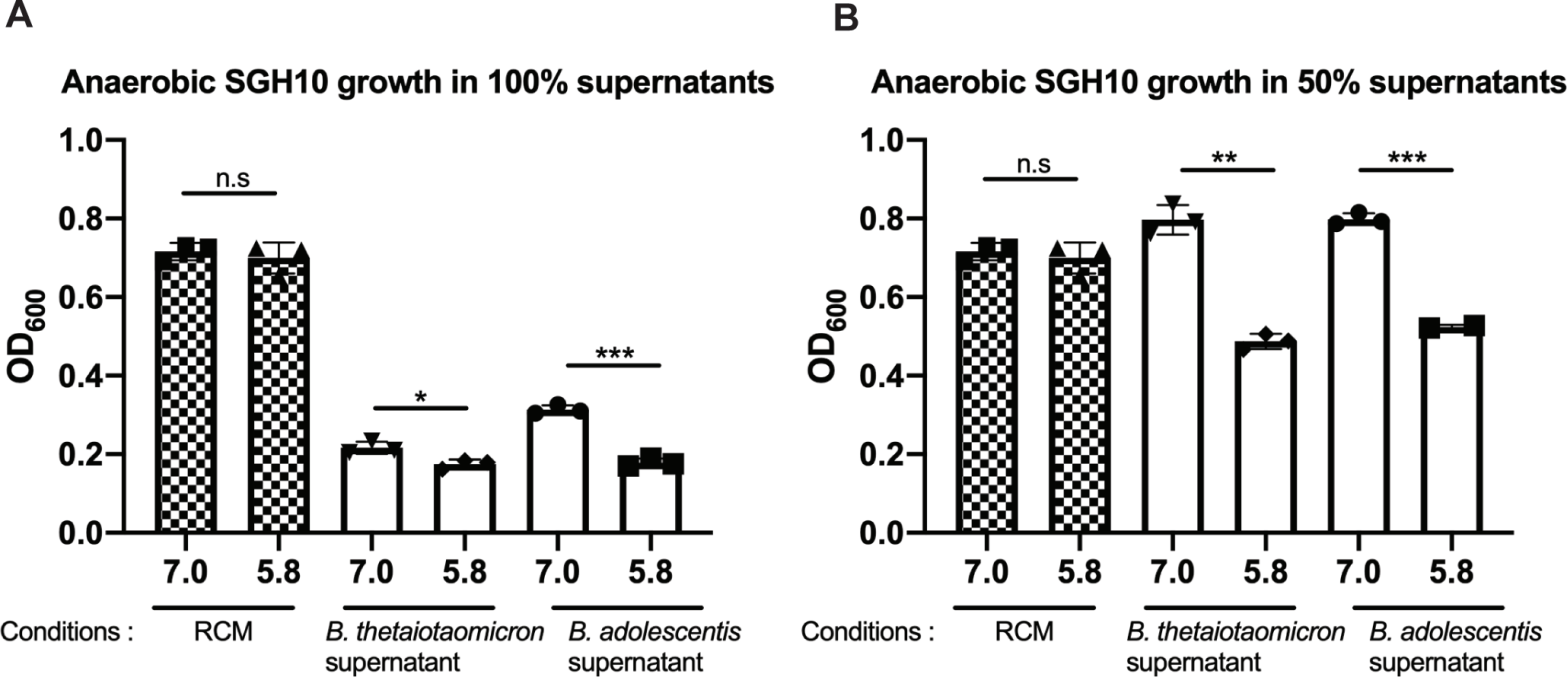
Anaerobic 48-hour OD_600_ growth of *K. pneumoniae* SGH10 in pH 7.0 and pH 5.75 of (**A**) 100% filtered supernatants and (**B**) 50% filtered supernatants of *B. thetaiotaomicron* and *B. adolescentis*. Media-only (RCM) pH-adjusted controls were included. Independent experiments have been repeated with similar observations.

Although there was no direct inhibitory effect of the anaerobes on SGH10 on solid media, SGH10 grown anaerobically in spent liquid supernatants (100%[vol/vol]) from *B. thetaiotaomicron* and *B. adolescentis* exhibited significant growth inhibition (Fig. 2A). The growth inhibition was completely abrogated upon decreasing the supernatant concentration from both anaerobes to 50% at pH 7.0 (Fig. 2B). Growth was still inhibited in 50% supernatants at pH 5.8 when compared to the media control at pH 5.8. Analysis of the anaerobic supernatants via GC-MS revealed high concentrations of acetate at a range of 141.05 to 221 mmol/L (Table 2).

**TABLE 2:** SCFA measurements via GC-MS of supernatants from *B. thetaiotaomicron* and *B. adolescentis* grown anaerobically in RCM

SCFA	Mean ± SD (mM)	
	*B. thetaiotaomicron*	*B. adolescentis*
Acetic acid	182.58 ± 3.98	175.82 ± 40.99
Propionic acid	0.057 ± 0.0013	0.058 ± 0.013
Iso-butyric acid	0.054 ± 0.0017	0.057 ± 0.012
Butyric acid	0.067 ± 0.00074	0.069 ± 0.015
Isovaleric acid	0.0178 ± 0.00030	0.018 ± 0.004
Valeric acid	0.0017 ± 0.00023	0.0014 ± 0.00041
Caproic acid	0.0020 ± 0.00026	0.0017 ± 0.00040

### SCFAs differentially inhibit *Enterobacteriaceae* at physiological pH

The gut microbiota is enriched in SCFA species such as acetate (C2), propionate (C3), and butyrate (C4). These are released upon fermentation of monosaccharides when dietary fiber is broken down by the gut microbiota in the lower colonic gastrointestinal tract in the molar ratio of 60:20:20 (29). The cecum is an intraperitoneal pouch marking the first entry point to the large intestine. It harbors the main reservoir of gut microbiota species and is the primary site of extensive fermentation and, hence, slightly acidic in nature (30). Studies measuring mouse cecum pH have reported it to be 5.94 (31), while human studies have reported the cecum and ascending colonic pH to be 5.7 before gradually increasing to pH 6–7 in the transverse and descending colon (32).

**Fig 3:**
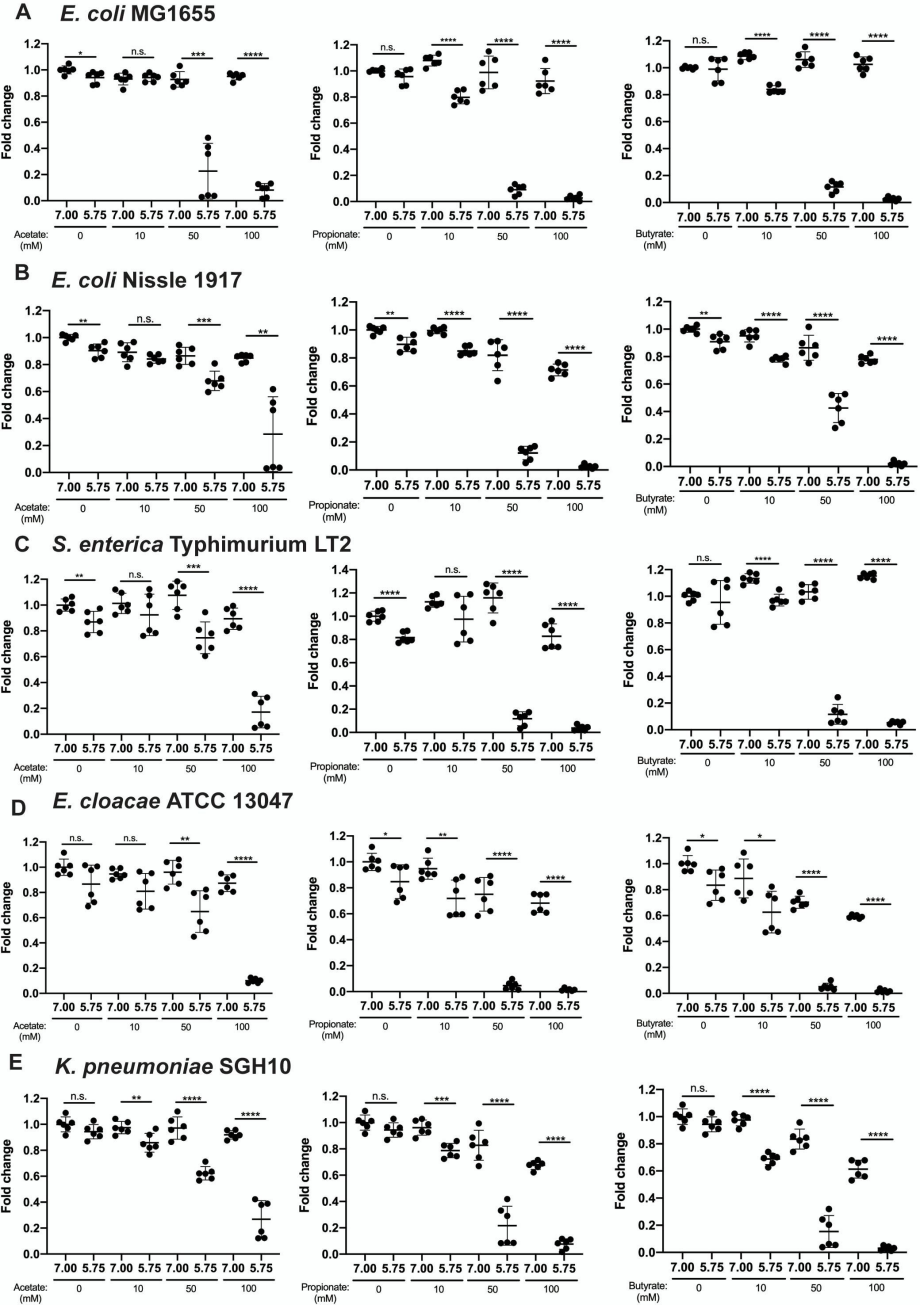
Aerobic 24-hour OD_600_ growth of (**A**) *E. coli* MG1655, (**B**) *E. coli* (Nissle 1917), (**C**) *S. enterica* Typhimurium LT2, (**D**) *E. cloacae* ATCC13047, and (**E**) *K. pneumoniae* SGH10 grown in the presence of 10, 50, and 100 mM of LB containing sodium acetate, propionate, and butyrate, pH-adjusted to pH 7.0 and 5.75. SCFA-free LB-only pH-adjusted controls were included for all experiments. Data are presented as mean ± SD of fold change of 24-hour OD_600_ relative to pH 7.0 0-mM SCFA control from two independent experiments. Welch’s *t*-test was used for statistical analysis; *P value < 0.05, **P value < 0.01, ***P value < 0.001, ****P value < 0.0001.

*Enterobacteriaceae* species including the intestinal commensals *E. coli* MG1655, *E. coli* (Nissle 1917; EcN), gut pathogens hypervirulent *K. pneumoniae* SGH10 and *S. enterica* Typhimurium, and the opportunistic nosocomial pathogen *E. cloacae* were inhibited by high concentrations of all three SCFAs acetate, propionate, and butyrate at cecum and ascending colonic pH 5.75 (Fig. 3). In the absence of SCFAs at pH 5.75, there was little to no growth inhibition on the bacterial strains relative to pH 7.0, demonstrating that acidic pH alone is not the critical factor required for bacterial inhibition. Supernatant measurements show that acetate is strongly inhibitory between 100 and 200 mM (Table 2). Thus, the SCFA concentrations were titrated down from 100, 50, to 10 mM to determine the effective concentration for inhibition. At 100 mM concentrations of all three SCFAs, all bacterial strains were maximally inhibited at pH 5.75. However, at concentrations below 100 mM, there were observable SCFA and strain-specific variations in inhibition.

We found that 50 mM acetate partially inhibited all strains at pH 5.75; whereas it inhibited *E. coli* MG1655 almost maximally (Fig. 3A). All other strains (Fig. 3B through D) were only maximally inhibited at 100 mM acetate at pH 5.75, except for SGH10 and EcN (Fig. 3B and E). Propionate was more inhibitory than acetate, where 50 mM at pH 5.75 maximally inhibited all strains (Fig. 3A through D), except SGH10, which was only maximally inhibited at 100 mM at pH 5.75 (Fig. 3E). Similarly, butyrate was more inhibitory than acetate, where 50 mM butyrate at pH 5.75 maximally inhibited *E. coli* MG1655, *S. enterica*, and *E. cloacae* (Fig. 3A, C, and D), except EcN (Fig. 3B) and SGH10 (Fig. 3E), which were only maximally inhibited at 100 mM butyrate at pH 5.75.

### Anaerobic conditions potentiate low pH-mediated SCFA inhibition of *K. pneumoniae*

**Fig 4:**
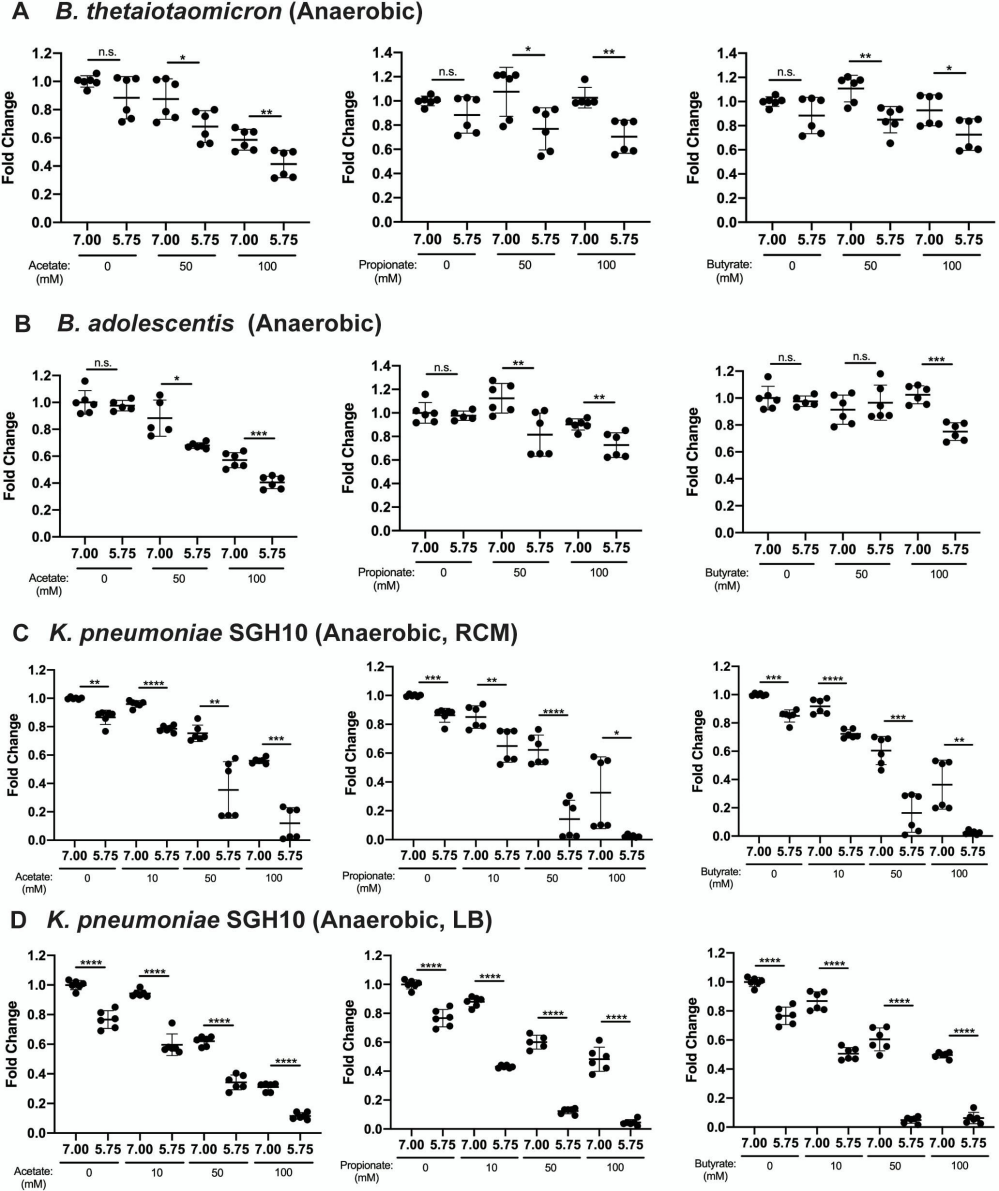
Anaerobic 48-hour OD_600_ growth of (**A**) *B. thetaiotaomicron,* (**B**) *B. adolescentis*, (**C**) *K. pneumoniae* SGH10 grown in the presence of 10, 50, and 100 mM of SCFAs in RCM or LB (**D**) containing sodium acetate, propionate, and butyrate, pH-adjusted to 7.0 and 5.75. SCFA-free RCM and LB-only pH-adjusted controls were included for all experiments. Data are presented as mean ± SD of fold change of 48-hour OD_600_ relative to pH 7.0 0 mM SCFA control from two independent experiments. Welch’s *t*-test was used for statistical analysis; *P value < 0.05, **P value < 0.01, ***P value < 0.001, ****P value < 0.0001.

There is a steep oxygen gradient that spans across the healthy GI tract. The colon is a largely anaerobic environment with very low oxygen concentration to support the growth of obligate anaerobes. Thus, it is necessary to ascertain whether cecum and colonic pH-mediated SCFA inhibition occurs in these anaerobic environments. It is also important to determine whether anaerobic gut commensals are inhibited by SCFAs as they would likely have adapted to this environment. Thus, *B. thetaiotaomicron*, *B. adolescentis*, and *K. pneumoniae* SGH10 were grown in the presence of the three SCFAs in anaerobic conditions. Inhibition of commensals increased with acetate concentration even at pH 7.0, while 50 mM propionate and butyrate were slightly inhibitory only at pH 5.75 (Fig. 4A and B). In contrast, SGH10 was severely inhibited by all SCFAs (Fig. 4C and D). Although 50 mM acetate and propionate at pH 5.75 only partially inhibited SGH10 aerobically (Fig. 3E), they severely inhibited its growth anaerobically (Fig. 4C and D). SGH10 was also anaerobically inhibited at pH 7.0 with increasing SCFA concentrations.

### SCFAs inhibit growth dynamics differently but reduce membrane potential similarly

SCFAs are uncharged weak acids with similar p*K*a values. The neutral, protonated acid (HA) form diffuses across the cell membrane to then dissociate into protons and anions. The lower the environmental pH, the more undissociated neutral HA form enters through the bacterial membrane for subsequent dissociation. Proton influx is commonly associated with an increase in intracellular pH if acid tolerance and stress response systems are overwhelmed, as well as disrupting proton motive force (PMF) consisting of transmembrane electric potential (ΔΨ) and intracellular pH (ΔpH). To probe potential mechanisms for the differential strengths of inhibition by the SCFAs, we looked at their effect on membrane potential ΔΨ using the fluorescent probe diethyloxacarbocyanine [DiOC_2_(3)], commonly used for measuring membrane potential in bacteria. At low concentrations, the dye exhibits a green fluorescence in all bacterial cells, but becomes more concentrated in healthy cells that are maintaining a membrane potential, causing the dye to self-associate and the fluorescence emission to shift to red. Membrane potential is, thus, expressed as the ratio of red/green fluorescence emission ratios.

**Fig 5:**
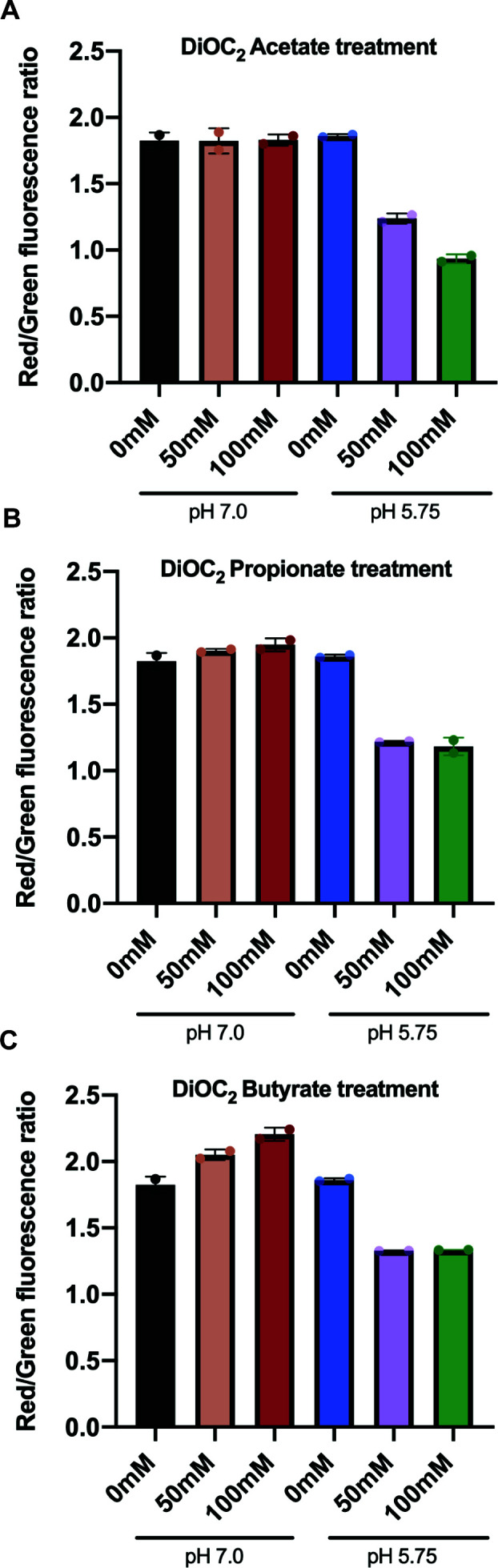
Membrane potential expressed as red/green fluorescence ratio (λ_EM_ 630 nm/λ_EM_ 530 nm) of SGH10 stained with DiOC_2_(3) dye after 2 hours of treatment with 50 and 100 mM of (**A**) sodium acetate, (**B**) propionate, and (**C**) butyrate at pH 7.0 and pH 5.75. SCFA-free pH-adjusted 1X PBS containing 0.4% glucose was included as controls. Data are presented as mean ± SD of biological triplicates. Independent experiments have been repeated with similar observations. Tukey’s multi-comparison test was used for statistical analysis. ***P value < 0.001, ****P value < 0.0001.

Membrane potential was maintained in SGH10 at pH 7.0 and 5.75 in the absence of SCFAs. There was no significant difference in membrane potential after 2 hours of SCFA treatment at pH 7.0. However, there was a significant decrease in membrane potential under SCFA treatment at pH 5.75 (Fig. 5), suggesting that physiological concentrations of SCFAs at cecum and ascending colon pH compromise the maintenance of a healthy membrane potential in bacteria. The reduction in membrane potential, however, does not appear to have a dose-dependent response to SCFA concentrations as 50 mM and 100 mM SCFA at pH 5.75 have similar membrane potential ratios. The degree of reduction in membrane potential of SGH10 at pH 5.75 by all three SCFAs versus in the SCFA-free control was also similar, suggesting that the differential growth inhibition by different SCFAs is not due to variations in membrane potential.

### SCFAs are bacteriostatic

**Fig 6:**
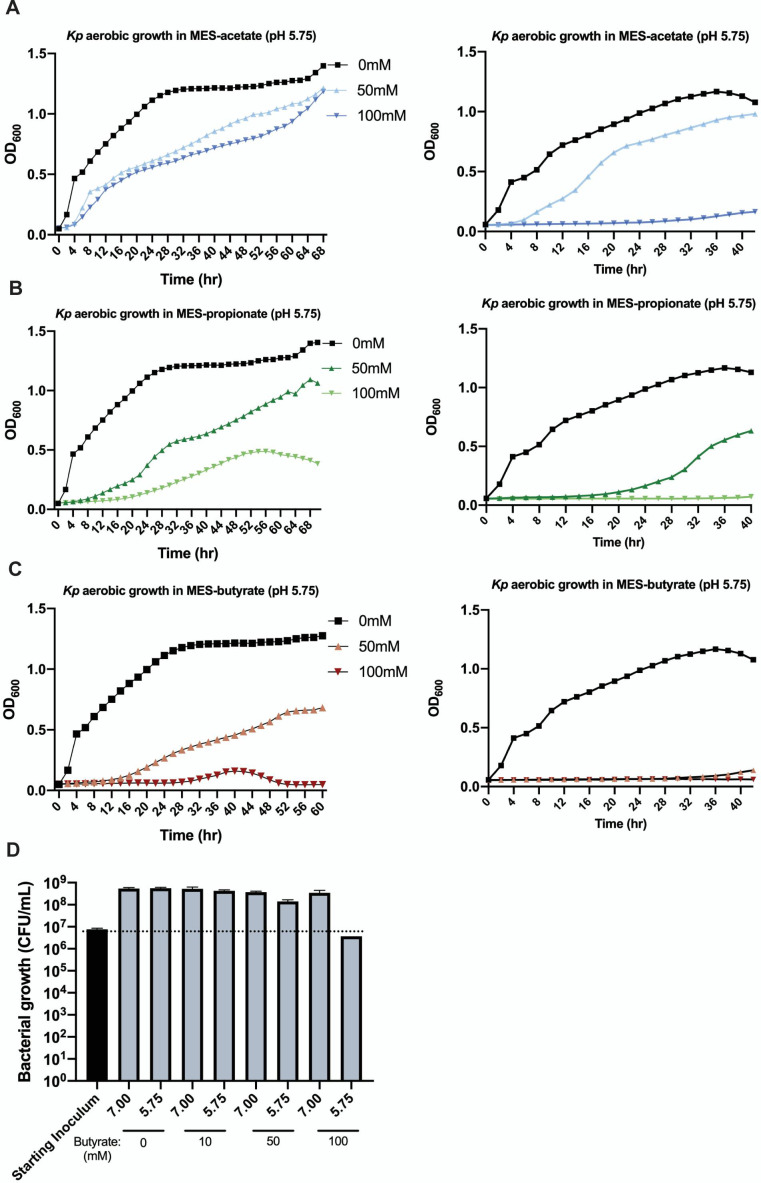
Growth curves from *K. pneumoniae* SGH10 grown at pH 5.75 in LB and LB + (**A**) sodium acetate, (**B**) sodium propionate, and (**C**) sodium butyrate. (**D**) Bacterial counts of *K. pneumoniae* SGH10 grown aerobically for 24 hours in LB and LB + 10, 50, and 100 mM of sodium butyrate at pH 7.0 and pH 5.75 to enumerate bacterial counts. Data are presented as mean ± SD of triplicates of two independent experiments (left and right columns).

We then determined whether SCFA inhibition of bacteria persists beyond 24 hours. SGH10 was grown in pH 5.75 in SCFA-free LB and LB with 50 and 100 mM of the three SCFAs. MES buffer (100 mM) was added to all growth conditions to maintain a constant pH over the extended incubation duration. We found differential effects of the SCFAs on growth. Fifty and 100 mM acetate extended and delayed the lag phase of growth, but eventually, SGH10 was able to reach similar levels of maximal carrying capacity or growth in 50 mM acetate as the SCFA-free control (Fig. 6A). SGH10 recovered partial growth capacity in 50 mM propionate over time (Fig. 6B), while butyrate at 50 mM still demonstrated sustained inhibition (Fig. 6C). One hundred millimolars of propionate and butyrate was strongly inhibitory, where they not only extended the lag phase but also confer sustained inhibition over time to prevent the attainment of maximal carrying capacity. One hundred-millimolar butyrate also causes much more persistent and drastic growth inhibition than 100 mM propionate, where there was no observable growth for the first 32 hours (Fig. 6C). To determine if the SCFAs were bacteriostatic or bactericidal, bacterial counts were enumerated at the start and 24 hours after SGH10 was inoculated into SCFA-free LB and LB with 10, 50, and 100 mM of sodium butyrate at pH 5.75 and 7.0. SCFAs appear to be bacteriostatic rather than bactericidal, as bacterial numbers in the strongest inhibitory condition of 100 mM butyrate at pH 5.75 are the same as the starting inoculum (Fig. 6D).

### Butyrate family acids are strongly inhibitory and degree of inhibition increases with alkyl chain length

**Fig 7:**
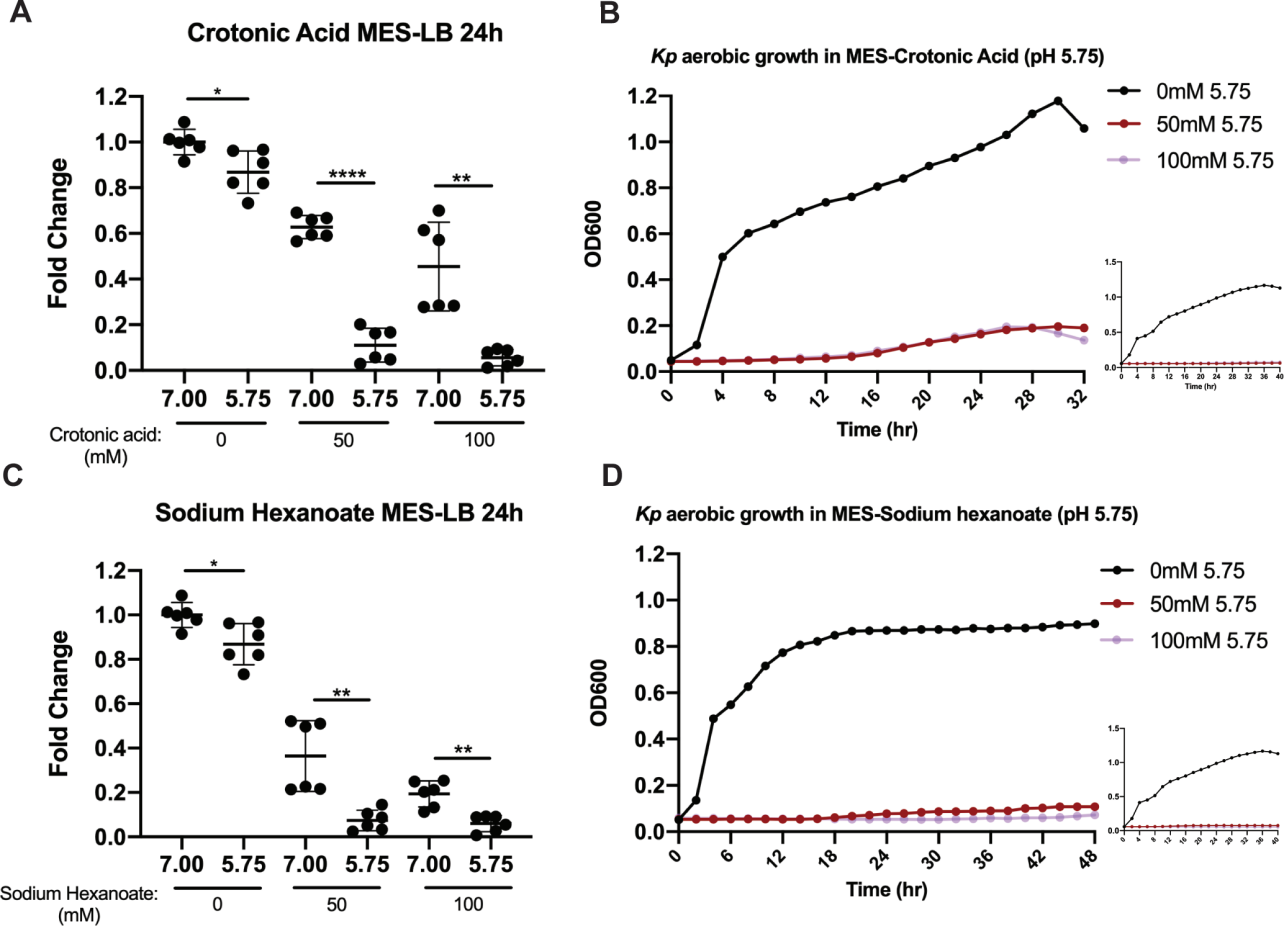
Aerobic OD_600_ 24-hour growth of SGH10 grown in LB containing 100 mM MES buffer at pH 7.0 and 5.75 with and without 50 and 100 mM of (**A**) crotonic acid, (**C**) sodium hexanoate, and the respective growth curves from two independent experiments over time (**B and D**) taken with a microplate reader. Data are presented as mean ± SD of fold change of 24-hour OD_600_ relative to pH 7.0 0 mM SCFA control from two independent experiments (inset shows the independent experimental repeat). Welch’s *t*-test was used for statistical analysis; *P value < 0.05, **P value < 0.01, ***P value < 0.001, ****P value < 0.0001.

As butyrate has the greatest inhibitory effects (Fig. 6), we wondered whether other C4 acids conferred the same extent of inhibition. In order to control for dissociative rates, we chose trans-2-butenoic acid or crotonic acid (C_4_H_6_O_2_ or CH_3_-CH=CH-COOH, p*K*a: 4.69) as it is a C4 butyrate family derivative, which shares a very similar p*K*a to butyrate (p*K*a: 4.82). Crotonic acid exhibited a very similar inhibition to that of butyrate (Fig. 7A and B), where it confers sustained inhibition over time at both 50 and 100 mM at pH 5.75, and also inhibited growth partially at pH 7.0 at these concentrations. The inhibitory strength of SCFAs increased from C2 (acetate) to C3 (propionate) and was strongest for C4 (butyrate), suggesting an alkyl-chain length-dependent increase in strength of inhibition. Therefore, we tested sodium hexanoate or sodium caproate (C_6_H_11_NaO_2_), which has a longer alkyl-chain acid with similar p*K*a (p*K*a = 4.88) to butyrate. The 24-hour endpoint fold changes were similar for crotonic acid and sodium hexanoate (Fig. 7A and C), and its inhibitory effect over time was even more pronounced, where SGH10 was completely inhibited throughout the entire duration of the experiment (Fig. 7B and D). This suggests that the degree of inhibition correlates with alkyl chain length.

## DISCUSSION

A healthy gut microbiota is instrumental in colonization resistance against pathobionts (1), where primary keystone species could potentially first establish niches for themselves and then provide subsequent cross-feeding networks to allow for the rapid succession of tertiary species (12). *B. thetaiotaomicron* is a proposed keystone species vital for ecological resilience due to its unique property of degrading host mucins under nutrient-deficient conditions to support the complex cross-feeding food-web in the gut and persisting in the face of pathogen challenge (14). We demonstrate that there are no direct inhibitory effects of three recovery-associated gut commensals against *K. pneumoniae* when co-cultured on solid media.

Although the gut commensals did not directly inhibit *K. pneumoniae* through close contact, their metabolic products strongly inhibited *K. pneumoniae*. Several groups have reported the intraluminal pH of the human lower gastrointestinal tract to be around 5.8 in the cecum and ascending colon (32); thus, the effect of the gut commensal supernatants on *K. pneumoniae* growth was examined at both neutral pH and physiological cecum pH. Our findings demonstrate a very strong inhibitory effect of the supernatants on anaerobic growth of *K. pneumoniae*. Metabolite measurements of the gut commensal supernatants reveal they are highly enriched in acetate at physiologically reported millimolar concentrations (29), strongly suggesting SCFA-mediated inhibition. Alternatively, inhibition could be due to nutrient depletion as *K. pneumoniae* may be unable to access certain nutrients efficiently in anaerobic conditions due to utilization of alternative electron acceptors.

In the gut, the three main SCFAs are acetate, propionate, and butyrate in the molar ratio of 60:20:20. SCFAs are at highest abundance in the distal intestine with reports of concentrations reaching up to 100 mM in human subjects (29). Majority of gut microbiota species are acetate producers, with only a handful of species capable of producing butyrate (33). Pathways for acetate production are commonly shared across multiple classes of bacteria (34), allowing it to be the dominant SCFA produced in the intestinal lumen. In contrast, propionate and butyrate productions are more species-specific. Propionate production occurs via three main pathways—the succinate pathway commonly utilized by *Bacteroidetes* and members of the Firmicutes phyla, the propanediol pathway commonly utilized by *Lachnospiraceae*, and the acrylate pathway utilized by the soil bacterium *Clostridium propinonicum* and members of the Negativicutes family (35).

Butyrate is implicated in regulation of mucosal immunity via antimicrobial peptides such as LL-37 cathelicidin (36), Treg cell activity, and IL-10 production (37). It also functions as the main energy source for colonocytes and maintains tight junctions in the gut to prevent pathogen invasion (38). Butyrate can be produced from dietary carbohydrates via the formation of two molecules of acetyl-coenzyme A (CoA) to form acetoacetyl-CoA, which is then converted to butyryl-CoA. Following this, there are two pathways leading to the final production of butyrate—the butyryl-CoA:acetate CoA transferase pathway and the butyrate kinase terminal enzyme pathway. The latter pathway is utilized by only a few butyrogenic taxa such as *Coprococcus comes* and *Coprococcus eutactus*, while the former pathway is utilized by the majority of butyrate producers in the gut such as *Faecalibacterium prausnitzii*, *Eubacterium rectale*, and *Eubacterium halli*. This process relies on metabolic cross-feeding and co-operation between gut microbiota species, where acetate producers provide exogenous acetate to butyrate producers, which exchange butyryl-CoA for acetate to derive acetyl-CoA and butyrate, catalyzed by the butryl-CoA:acetate CoA transferase enzyme (39).

Sorbara et al. demonstrated that SCFAs had a direct inhibitory effect on various antibiotic-resistant clinical isolates of *K. pneumoniae*, *Proteus mirabilis*, and *E. coli* at mouse cecum pH of 5.75 (31). Here, we investigated the effect of SCFAs, as well as their derivatives, on a larger panel of *Enterobacteriaceae* and anaerobic commensals to understand how wide-ranging this inhibitory effect is. Our findings reveal that SCFAs strongly inhibit multiple prevalent *Enterobacteriaceae* species. Interestingly, we did not observe similar SCFA inhibition on the gut commensal species tested, with SCFAs only having mild inhibitory effects on *B. thetaiotaomicron* and *B. adolescentis*. Catlett et al. previously reported that excess acetate supplementation increased *B. thetaiotaomicron* doubling time and inhibited its growth (40). Metabolic flux analyses revealed increasing acetate supplementation to just 10 mM suppressed overall secretion of multiple metabolites including succinate, formate, propionate, and glutathione in the entire interlinked metabolic network in the process of feedback inhibition to lower its own acetate production, affecting downstream amino acid synthesis pathways . This is in line with our finding that increasing acetate concentrations even at neutral pH confers inhibition on gut commensals. However, to the best of our knowledge, the mild inhibition conferred by propionate and butyrate has not been previously reported.

The lower GI tract is primarily devoid of oxygen and comprises trillions of resident anaerobes. These anaerobic conditions potentiate low pH-mediated SCFA inhibition of *K. pneumoniae*, where inhibition was observed even at neutral pH unlike in aerobic conditions. SGH10 also experienced severe growth inhibition at lower concentrations of SCFAs in anaerobic versus aerobic conditions. This is likely due to the collapse of proton motive force (Δp), which consists of transmembrane electrical potential (ΔΨ) and proton gradient (ΔpH) (41). In anaerobic conditions, electric potential is compromised so the predominant force for PMF generation relies on the ΔpH gradient. However, at pH 5.75, the ΔpH is also compromised. The absence of both ΔΨ and ΔpH causes a collapse in the PMF and downstream cellular processes such as ATP synthesis, nutrient import, and various metabolic processes important for bacterial growth, and metabolism reliant on PMF generation. SCFA accumulation could further impair fermentation and anaerobic metabolic pathways. This potentiation of SCFA inhibition under anaerobic conditions could also explain why the RAB supernatants were more strongly inhibitory at neutral pH in anaerobic rather than aerobic conditions.

SCFAs are neutral, uncharged weak acids with similar p*K*a values between 4.75 and 4.8. The neutral protonated form diffuses across the hydrophobic lipid bilayer of bacterial cell membranes before it dissociates into protons and anions within the neutral cytosolic compartment. Degree of deprotonation is dependent on the external environmental pH; although external pH 5.75 and pH 7.0 are higher than the p*K*a, there is significantly lesser degree of protonation (8.9 H+ protons for each HA molecule) and, therefore, higher amount of undissociated molecules of neutral acid at pH 5.75 versus at 7.0 (158 H+ protons for each HA molecule), which enters the bacterial cytosol for subsequent dissociation. This explains why the strongest inhibition of *Enterobacteriaceae* was under high SCFA concentrations at pH 5.75, not pH 7.0. The longer alkyl-chain SCFAs are strongly inhibitory at 50 mM in acidic pH except for Nissle 1917 and SGH10, which were only partially inhibited. Nissle 1917 is a resident intestinal commensal, while SGH10 is a successful enteric pathogen where its first point of entry is through the fecal-oral route before establishing colonization in the gut (42). We hypothesize that perhaps they have evolved to have more efficient proton removal pathways against acid stress and intracellular acidification, which makes them better adapted gut species that are able to persist in the face of SCFA dissociation in the circumneutral cytosol. The longer chain SCFAs may be capable of causing further intracellular acidification by directly acting on proton pumps to cause ineffective removal of cytosol proton accumulation in the other *Enterobacteriaceae*.

Upon SCFAs dissociating into protons and anions within the circumneutral bacterial cytoplasm, the strain-specific variations in inhibition are likely due to their ability to induce pleiotropic downstream cellular effects, which have previously been reported (43). Proton influx causes intracellular acidification, and the degree of proton perturbation and subsequent effects on PMF will depend on strain-specific acid tolerance responses (ATR) and the success of these ATR systems in eliminating cytosol proton accumulation. We have shown that SCFAs at pH 5.75 reduce membrane potential but do not appear to be dose-dependent on SCFA concentrations, as 50 and 100 mM SCFA at pH 5.75 have similar membrane potential ratios. This suggests that the stronger inhibition by longer chain SCFAs is not due to greater disruption of membrane potential and likely occurs via other mechanisms. Anion influx has been reported to cause differential effects on metabolic responses as these organic acid anions can act as metabolically disruptive carbon metabolites that feed into the tricarboxylic acid (TCA) cycle, also known as the Krebs cycle, or membrane fatty acid synthesis in some pathogens, disrupting amino acid uptake and transport and perturbing osmotic balance (44). DNA synthesis in *E. coli* has been shown to be more sensitive to propionate disruption than for synthesis of other cellular components such as proteins, RNAs, lipids, or cell walls (45). Acetate and propionate exposures were shown to inhibit amino acid uptake in *Bacillus subtilis,* while in contrast, acetate was shown to increase the transcript level of amino acid transporters in *E. coli*.

We have shown that at 100 mM concentrations, acetate delays *K. pneumoniae* growth but does not affect maximal growth capacity achieved over time, propionate prevents it from reaching maximal growth capacity, and butyrate completely suppresses growth. This strong bacteriostatic effect is also observed with a C4 family derivative crotonic acid and even more pronounced with C6 sodium hexanoate. This demonstrates an alkyl-length-dependent strength of inhibition independent of the rate of deprotonation as the p*K*a values of all C2–C6 acids in our study are between 4.75 and 4.88. This association between alkyl chain length and toxicity is in line with previous reports where the longer chain decanoic acid (C10) was maximally inhibitory at a lower concentration than hexanoic (C6) and octanoic (C8) acids against *E. coli* MG1655 (46), coupled with loss of membrane fluidity and integrity. Sensitivity to membrane fluidization, but not membrane integrity, was partially alleviated via short-term adaptation of MG1655 for 3 hours only to octanoic acid. This altered the saturated and unsaturated lipid ratio of outer membrane lipid structure to increase tolerance. Butyrate has been previously reported to alter membrane composition by assimilating into straight-chain fatty acids, which are found in small amounts in membrane fatty acids (47), eliciting a membrane-altering effect significantly different from other organic acids like hydrochloric acid, lactate, or acetate (48). The increased toxicity of C6 over the shorter-chain fatty acids could, thus, be due to its increased disruption of membrane fluidity and lipid rearrangement.

We have demonstrated a differential ability of SCFAs to inhibit the growth of multiple enteric pathogens, but less significantly for the two major gut commensals we tested, in a p*K*a and membrane potential-independent manner. Rather, inhibitory strength correlated with alkyl chain length perhaps via increased bacterial membrane destabilization. Importantly, prevalent CPE species *K. pneumoniae*, *E. cloacae*, and *E. coli* are effectively inhibited. This presents an avenue for potential new therapeutic treatments and possible feed additives as a colonization resistance or decolonization strategy against a wide range of enteric pathogens including multidrug-resistant (MDR) species. Dietary fiber supplementation allows for greater fermentation and SCFA production capability by gut microbiota. Although butyrate is the least abundant SCFA produced in the gut, cross-feeding of acetate to butyrate producers could enhance its production. Besides prebiotic supplementation to encourage *de novo* production of SCFA, an alternative therapeutic for an infection-permissive dysbiotic gut is the direct exogenous administration of longer alkyl chain length compounds. This strategy is more appropriate for dosing SCFAs such as the longer-chain fatty acids like caproic acid (C6), which are present at lower physiological gut concentrations than the three main SCFAs. Butyrate glycerides are created through the fusion of any variable number of butyrate molecules to a glycerol backbone, which protects it from absorption in the GI tract and only released upon intestinal lipase action (49). A similar encapsulated form of oral delivery for longer alkyl-chain acids can be adopted to allow for slow release in the lower GI tract. Taken together, fiber supplementation to enhance endogenous SCFA production as well as direct administration can maximize the antimicrobial function of SCFAs. This makes it a strong therapeutic alternative to certain bacteriostatic antibiotics to effectively inhibit MDR bacteria without exacerbating antimicrobial-resistance (AMR)-induced dysbiosis.
